# Pharmacological interventions for melanoma: Comparative analysis using bayesian meta-analysis

**DOI:** 10.18632/oncotarget.12644

**Published:** 2016-10-13

**Authors:** Yang Yang, Jiaomiao Pei, Guozhen Gao, Zheng Yang, Shuzhong Guo, Bo Yue, Jianhua Qiu

**Affiliations:** ^1^ Department of Plastic and Reconstructive Surgery, Xijing Hospital, Fourth Military Medical University, Xi'an, 710032, China; ^2^ Medical Insurance and New Rural Cooperative Medical Insurance Administration Center, The 252nd Hospital of PLA, Baoding, 071000, China; ^3^ Department of Burn and Plastic Surgery, The 253rd Hospital of PLA, Hohhot, 010051, China; ^4^ The First Brigade of Fourth Military Medical University, Xi'an, 710032, China; ^5^ Department of Otolaryngology-Head and Neck Surgery, Xijing Hospital, Fourth Military Medical University, Xi'an, 710032, China

**Keywords:** melanoma, chemotherapy, ipilimumab, tremelimumab, nivolumab

## Abstract

We conducted a network meta-analysis in order to compare different strategies for managing melanoma patients. Electronic databases were searched for eligible randomized trials that compared different strategies in efficacy and tolerability. Five interventions were associated with a significant improvement in PFS over chemotherapy (all HR < 1): Ipilimumab, Tremelimumab, Nivolumab, Pembrolizumab 2 mg/kg and Ipilimumab + Nivolumab. Three interventions exhibited significantly improved OS results over chemotherapy (all HR < 1): Ipilimumab, Nivolumab and Ipilimumab + Chemotherapy. Four interventions were superior to chemotherapy in CR and PR (all OR > 1): Nivolumab, Pembrolizumab 10 mg/kg, Pembrolizumab 2 mg/kg and Ipilimumab + Nivolumab. However, the other seven interventions were associated with an increased risk of pruritus compared to chemotherapy (all OR > 1). Ipilimumab, Tremelimumab, Ipilimumab + Nivolumab and Ipilimumab + Chemotherapy might result in a higher risk of diarrhea compared to chemotherapy (all OR > 1). Immune checkpoint therapy or combined interventions might be more effective than chemotherapy for managing melanoma patients. However, chemotherapy appears to be more tolerable than these combined strategies with respect to adverse events.

## INTRODUCTION

Melanoma is a tumor due to melanocytes that develop in various areas such as skin, mucosal membranes, eyes and meninges. It was estimated that 100,000 new melanoma cases were diagnosed in 2012 and the expected number of deaths due to the progression of skin cancer was about 22,000 in 2012 [1]. Despite of the fact that a wide range of therapies have been developed, the prognosis of melanoma is not optimistic since patients tend to have poor responses to traditional treatments such as chemotherapy and radiation therapy [2]. In order to achieve improvement, new approaches have been advocated.

Chemotherapy is usually involved in the systemic treatment of melanoma but it has limited effectiveness [3]. On the other hand, a number of immunotherapy and targeted therapy agents have been authorized to improve the survival status of melanoma patients [4]. Antibodies have been introduced into clinical practices to stimulate the immune system by enhancing anti-tumor responses [5]. Ipilimumab is an antibody able to block the co-inhibitory receptor cytotoxic T-lymphocyte antigen-4 (CTLA-4) and the inhibition of CTLA-4 contributes to a global activation of the immune system and thereby improving the survival status of melanoma patients [5–7]. Emerging data have suggested that synergetic effects can be generated by combining two different treatment strategies simultaneously [8]. For instance, an abscopal effect has been demonstrated by introducing both radiation and Ipilimumab [9]. Besides that, combining chemotherapy with checkpoint inhibitors such as Ipilimumab represents a novel way to optimize the corresponding effect of checkpoint inhibitors on melanoma patients. The success of Ipilimumab has triggered the development of other immune-modulating antibodies.

The use of Tremelimumab as an immune checkpoint therapy is still in progress, but it has exhibited some effectiveness in metastatic melanoma and other cancers [10]. As suggested by Canniff et al., the effect of Tremelimumab does not depend on the disease stage or cancer type and Tremelimumab is able to enhance the production of IL-2 in T-cells among both healthy controls and cancer patients with solid tumors [10]. However, it appears that Tremelimumab does not exhibit compelling results in phase III trials and hence potential factors that have significant influence on Tremelimumab plasma exposure should be further investigated [11]. Nivolumab is another immune checkpoint inhibitor approved for managing metastatic melanoma, squamous cell lung cancer, and renal cell cancer [12]. Although Nivolumab is generally well-tolerated, some melanoma patients experienced severe pneumonitis after receiving Nivolumab treatment [12]. Moreover, combining different immune checkpoint inhibitors such as Ipilimumab and Nivolumab has been approved by FDA recently and the combined strategy appeared to outperform each monotherapy with respect to response rate and PFS [13]. However, the major drawbacks of using these checkpoint inhibitors simultaneously are the increased level of toxicity and more adverse events such as rash, pneumonitis, diarrhea and colitis [14].

Apart from CTLA-4, programmed death 1 (PD-1) is another well-known immune checkpoint protein which exhibits distinctive mechanisms in cells [15]. Unlike CTLA-4, the PD-1 pathways are able to regulate immune responses in tissues selectively [16] and hence several antibodies targeting PD-1 have been developed in various clinical stages. Pembrolizumab is a humanized monoclonal antibody that inhibits the interaction between PD-1 on T cells, thereby triggering antitumor immune responses related to the PD-1 pathways [15]. As suggested by a randomized cohort study in which patients experienced Ipilimumab-refractory melanoma, the objective response rate (26%) is equivalent for those who received Pembrolizumab at the dosage of 2 mg/kg or 10 mg/kg every three weeks [17]. One strength of Pembrolizumab is that it is well-tolerated without clear evidence of increased toxicity due to the increase in dosage [18]. However, comparing the efficacy and tolerability between Pembrolizumab and Nivolumab is challenging since they were assessed in different patient populations and it is also very hard to determine the optimal treatment duration for Pembrolizumab [15].

Since the number of immune checkpoint inhibitors approved by FDA is increasing, it is critical to differentiate those inhibitors with respect to their efficacy and safety. Our research was inspired by the rapid development and aimed to provide consistent evidence for the selection of interventions. There were two phases in this study. In the first phase, we searched for all the articles about pharmacological interventions introduced to melanoma. In the second phase, we incorporated the approach of network meta-analysis (NMA) to assess the relative efficacy and safety of the following selected interventions: chemotherapy, Ipilimumab, Tremelimumab, Nivolumab, Pembrolizumab (10 mg/kg or 2mg/kg), Ipilimumab + Nivolumab and Ipilimumab + Chemotherapy.

## RESULTS

### Literature search and study characteristics

**Figure 1:**
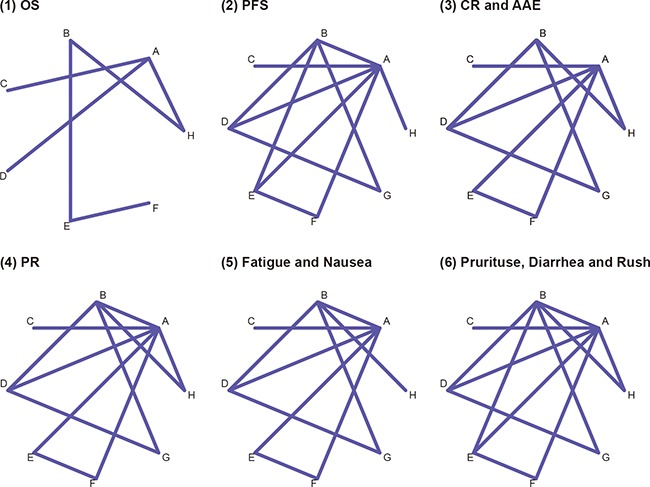
Network diagram Each node represents a melanoma therapy.

After ineligible and duplicated studies were removed, 19 studies were incorporated into the analysis with a total sample size of 6,405 subjects [17, 19–36]. All of these included studies were carried out between 2005 and 2015. Information of studies and clinical features of subjects were listed in Table 1. The Jadad scale results of methodological quality were presented in Supplementary Table S1. The comparison networks of interventions for each endpoint were illustrated in Figure 1.

**Table 1:** List of clinical trials testing the use of monoclonal antibodies in melanoma

Study	Trial ID	Phase	Intervention	Dosage (mg/kg)	N	Age yrs (mean, SD)	Male,%	Metastasis (n, %)		Outcomes
								M0-M1b	M1c	
Weber, 2015, USA	NCT01721746	III	Nibolumab	3	272	59(16)	176(65)		203(75)	⊠⊠⊠⊠⊠⊠⊠⊠
			Chemotherapy		133	62(14)	85(64)		102(77)	
Robert, 2015, France	NCT01866319	III	Pembrolizumab	10	279	61(18)	161(58)	94(35)	179(65)	⊠⊠⊠⊠⊠⊠
			Pembrolizumab	10	277	63(17)	174(63)	84(32)	189(68)	
			Ipilimumab	3	278	62(18)	162(58)	96(35)	177(65)	
Robert, 2015, France	NCT01721772	III	Nivolumab	3	210	64(17)	121(58)	82(39)	126(61)	⊠⊠⊠⊠⊠⊠⊠⊠⊠⊠
			Chemotherapy		208	66(15)	125(60)	81(39)	127(61)	
Ribas, 2015, USA	NCT01704287	II	Pembrolizumab	2	180	62(18)	104(58)	32(17)	148(83)	⊠⊠⊠⊠⊠⊠⊠⊠⊠
			Pembrolizumab	10	181	60(16)	109(60)	31(17)	150(83)	
			Chemotherapy		179	63(15)	114(64)	32(17)	147(83)	
Postow, 2015, USA	NCT01927419	III/IV	Ipilimumab+Nivolumab	3+1	72	64(15)	63(66)	50(53)	44(47)	⊠⊠⊠⊠⊠⊠⊠⊠⊠
			Ipilimumab	3	37	67(12)	32(68)	25(55)	21(45)	
Miao, 2015, Italy	CA184-024	III	Ipilimumab+Chemotherapy	10	40	58(13)	23(62)	22(55)	18(45)	⊠⊠
			Chemotherapy		20	61(11)	9(45)	15(75)	5(25)	
Larkin, 2015, USA	NCT01844505	III	Nivolumab	3	316	59(16)	202(64)	312(42)	184(58)	⊠⊠⊠⊠⊠⊠⊠⊠⊠
			Ipilimumab+Nivolumab	3+1	314	59(18)	206(66)	133(42)	181(58)	
			Ipilimumab	3	315	61(18)	202(64)	132(42)	183(58)	
Eggmont, 2015, France	NCT00636168	III	Ipilimumab		475	51(16)	296(62)			⊠⊠⊠⊠⊠⊠⊠
			Chemotherapy		476	52(15)	293(62)			
Robert, 2014, France	NCT01295827	I	Pembrolizumab	2	89	57(17)	48(54)	31(35)	58(65)	⊠⊠⊠⊠⊠⊠⊠
			Pembrolizumab	10	84	61(15)	57(68)	32(38)	52(62)	
Hodi, 2014, USA	NCT01134614	III/IV	Ipilimumab+Chemotherapy		123	61(15)	85(69)	33(27)	61(50)	⊠⊠⊠⊠⊠⊠⊠⊠
			Ipilimumab		122	64(17)	78(64)	31(25)	60(49)	
Ribas, 2013, USA	NCT00257205	III	Tremelimumab	15	328	57(17)	190(58)	121(37)	188(57)	⊠⊠⊠⊠⊠⊠⊠⊠⊠⊠
			Chemotherapy		327	56(17)	182(56)	119(36)	194(59)	
Millward, 2013, Australia		IV	Tremelimumab	6	3		14(67)	8(38)	9(43)	⊠⊠⊠⊠⊠⊠
			Tremelimumab	10	6					
			Tremelimumab	15	6					
Robert, 2011, France	NCT00324155	III/IV	Ipilimumab+Chemotherapy	10	250	58	152(61)	107(43)	143(57)	⊠⊠⊠⊠⊠⊠⊠⊠
			Chemotherapy		252	56	149(59)	113(45)	139(55)	
Hersh, 2011, USA	NCT00050102	II	Ipilimumab	3	40	66(14)	21(57)	16(43)	21(57)	⊠⊠⊠⊠⊠⊠⊠
			Ipilimumab+Chemotherapy	3	36	60(14)	26(74)	18(51)	16(46)	
Hamid, 2011, USA	NCT00261365	II	Ipilimumab	3	40	54(14)	28(70)	18(45)	22(55)	⊠⊠
			Ipilimumab	10	42	56(15)	24(57)	14(33)	28(67)	
Wolchok, 2011, USA	NCT00289640	III/IV	Ipilimumab	10	73	59(15)	52(71)	28(39)	45(62)	⊠⊠⊠⊠⊠⊠⊠⊠
			Ipilimumab	3	72	59(12)	48(67)	36(50)	36(50)	
Weber, 2009, UK		III/IV	Ipilimumab+Chemotherapy	10	58	58(13)	43(74)	30(52)	28(48)	⊠⊠⊠
			Ipilimumab	10	57	61(16)	38(67)	28(49)	29(51)	
Camacho, 2009, Finland	NCT0086489	I	Tremelimumab	6	3					⊠⊠⊠⊠⊠⊠⊠⊠
			Tremelimumab	10	22					
		II	Tremelimumab	10	44	61(15)	26(59)	14(32)	29(66)	
			Tremelimumab	15	46	54(16)	30(65)	11(25)	32(73)	
Ribas, 2005, USA	-	I	Ipilimumab	3	9	54(12)	27(69)			⊠⊠⊠
			Ipilimumab	10	11					

Metastasis Category: M0=no distant metastasis; M1a=metastasis to skin, subcutaneous tissues, or distant lymph nodes; M1b=metastasis to lung; M1c=to all other visceral sites or distant metastases; Outcome ⊠ PFS; ⊠ OS; ⊠ Complete Rate;⊠ Partial Rate; ⊠ All adverse events; ⊠ Fatigue; ⊠ Pruritus; ⊠ Rash; ⊠ Diarrhea; ⊠ Nausea.

### Comparison of effectiveness among melanoma interventions

**Figure 2:**
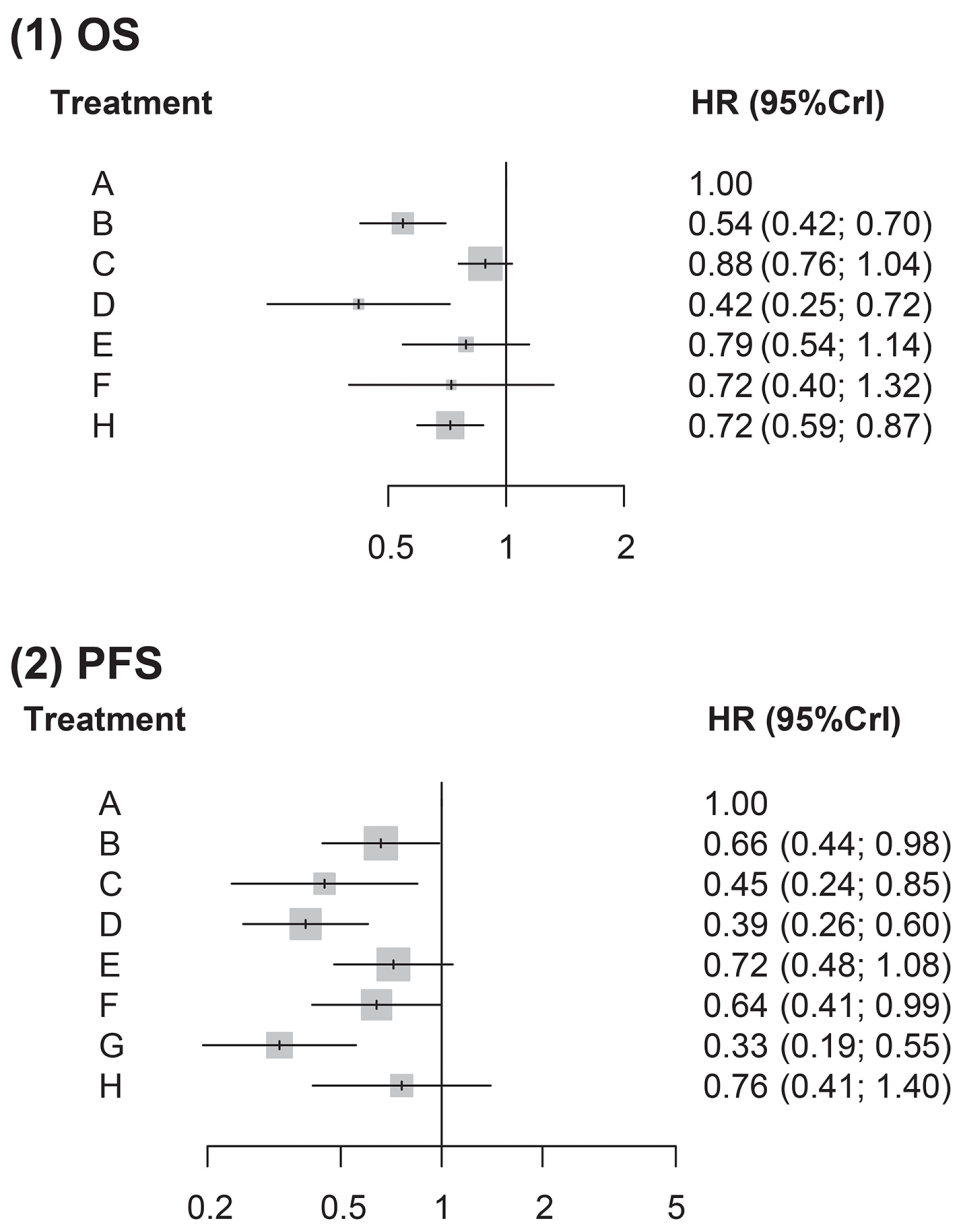
Forest plots for mixed treatment comparison of prognostic data overall survival & progression-free survival

Five interventions appeared to be more effective than chemotherapy with respect to PFS. For instance, patients treated with Ipilimumab were associated with an average 34% reduction in the HR of PFS compared with those treated with chemotherapy (HR = 0.66, 95% CrI = 0.44-0.98). The same trend were found in Tremelimumab (HR = 0.45, 95% CrI = 0.24-0.85), Nivolumab (HR = 0.39, 95% CrI = 0.26-0.60), Pembrolizumab 2 mg/kg (HR = 0.64, 95% CrI = 0.41-0.99) and Ipilimumab + Nivolumab (HR = 0.33, 95% CrI = 0.19-0.55). Furthermore, Nivolumab and Ipilimumab + Nivolumab were more effective than Ipilimumab (HR = 0.60, 95% CrI = 0.39-0.90; HR = 0.50, 95% CrI = 0.33-0.76) with respect to PFS. Patients treated with Ipilimumab + Nivolumab were associated with a decrease in the HR of PFS compared to those treated with Pembrolizumab 10 mg/kg (HR = 0.46, 95% CrI = 0.25-0.82) or Pembrolizumab 2 mg/kg (HR = 0.51, 95% CrI = 0.27-0.97). Finally, increasing the dose of Pembrolizumab appeared to have no significant effect on the HR of melanoma patients with respect to PFS (HR = 0.89, 95% CrI = 0.63-1.26; Table 2, Figure 2).

**Table 2:** Main NMA outcomes for melanoma

**PFS**	**A**	1.52 (1.02, 2.27)	2.24 (1.18, 4.23)	2.55 (1.66, 3.91)	1.39 (0.93, 2.09)	1.56 (1.01, 2.44)	3.05 (1.8, 5.16)	1.32 (0.71, 2.42)
	**0.66 (0.44, 0.98)**	**B**	1.47 (0.69, 3.13)	1.68 (1.11, 2.53)	0.92 (0.58, 1.44)	1.03 (0.61, 1.73)	2.01 (1.32, 3.05)	0.87 (0.42, 1.80)
	**0.45 (0.24, 0.85)**	0.68 (0.32, 1.44)	**C**	1.14 (0.53, 2.46)	0.62 (0.29, 1.33)	0.7 (0.32, 1.52)	1.36 (0.6, 3.12)	0.59 (0.24, 1.42)
	**0.39 (0.26, 0.6)**	**0.60 (0.39, 0.90)**	0.88 (0.41, 1.89)	**D**	0.55 (0.32, 0.93)	0.61 (0.35, 1.09)	1.2 (0.76, 1.88)	0.52 (0.24, 1.09)
	0.72 (0.48, 1.08)	1.09 (0.69, 1.72)	1.61 (0.75, 3.42)	**1.83 (1.07, 3.12)**	**E**	1.12 (0.79, 1.59)	2.19 (1.22, 3.93)	0.94 (0.45, 1.97)
	**0.64 (0.41, 0.99)**	0.97 (0.58, 1.63)	1.43 (0.66, 3.11)	1.63 (0.92, 2.9)	0.89 (0.63, 1.26)	**F**	1.95 (1.03, 3.67)	0.84 (0.40, 1.79)
	**0.33 (0.19, 0.55)**	**0.5 (0.33, 0.76)**	0.73 (0.32, 1.68)	0.84 (0.53, 1.31)	**0.46 (0.25, 0.82)**	**0.51 (0.27, 0.97)**	**G**	0.43 (0.19, 0.97)
	0.76 (0.41, 1.4)	1.15 (0.56, 2.40)	1.7 (0.7, 4.11)	1.94 (0.92, 4.09)	1.06 (0.51, 2.21)	1.19 (0.56, 2.53)	**2.32 (1.03, 5.19)**	**H**
**OS**	**A**	1.84 (1.43, 2.36)	1.13 (0.97, 1.32)	2.38 (1.39, 4.07)	1.27 (0.87, 1.84)	1.38 (0.76, 2.52)	-	1.39 (1.14, 1.69)
	**0.54 (0.42, 0.7)**	**B**	0.62 (0.46, 0.83)	1.3 (0.72, 2.34)	0.69 (0.52, 0.91)	0.75 (0.44, 1.30)	-	0.76 (0.65, 0.89)
	0.88 (0.76, 1.04)	**1.62 (1.21, 2.18)**	**C**	2.11 (1.21, 3.68)	1.12 (0.75, 1.68)	1.22 (0.66, 2.27)	-	1.23 (0.96, 1.58)
	**0.42 (0.25, 0.72)**	0.77 (0.43, 1.39)	**0.47 (0.27, 0.83)**	**D**	0.53 (0.28, 1.02)	0.58 (0.26, 1.30)	-	0.58 (0.33, 1.03)
	0.79 (0.54, 1.14)	**1.45 (1.1, 1.91)**	0.89 (0.6, 1.34)	1.88 (0.98, 3.61)	**E**	1.09 (0.68, 1.75)	-	1.1 (0.80, 1.51)
	0.72 (0.4, 1.32)	1.33 (0.77, 2.3)	0.82 (0.44, 1.52)	1.72 (0.77, 3.86)	0.92 (0.57, 1.47)	**F**	-	1.01 (0.57, 1.78)
	-	-	-	-	-	-	**G**	-
	**0.72 (0.59, 0.87)**	**1.32 (1.13, 1.55)**	0.81 (0.63, 1.04)	1.71 (0.97, 3.03)	0.91 (0.66, 1.25)	0.99 (0.56, 1.76)	-	**H**
**CR**	**A**	1.48 (0.31, 7.94)	1.32 (0.30, 6.45)	8.09 (2.28, 30.0)	7.36 (1.54, 37.9)	6.50 (1.31, 32.9)	**10.8 (2.29, 68.3)**	3.48 (0.82, 15.6)
	0.68 (0.13, 3.27)	**B**	0.89 (0.10, 8.66)	5.58 (1.61, 20.0)	4.98 (0.53, 50.1)	4.20 (0.43, 42.4)	7.43 (2.51, 27.3)	2.21 (0.46, 12.0)
	0.76 (0.16, 3.35)	1.12 (0.12, 10.03)	**C**	6.08 (0.76, 45.1)	5.77 (0.61, 49.1)	5.03 (0.51, 40.8)	8.28 (0.85, 86.2)	2.59 (0.31, 22.0)
	**0.12 (0.03, 0.44)**	**0.18 (0.05, 0.62)**	0.16 (0.02, 1.32)	**D**	0.94 (0.11, 7.13)	0.77 (0.10, 6.63)	1.34 (0.41, 5.14)	0.42 (0.08, 2.05)
	**0.14 (0.03, 0.65)**	0.20 (0.02, 1.89)	0.17 (0.02, 1.64)	1.06 (0.14, 9.07)	**E**	0.84 (0.25, 2.83)	1.46 (0.16, 16.5)	0.45 (0.05, 4.03)
	**0.15 (0.03, 0.76)**	0.24 (0.02, 2.31)	0.20 (0.02, 1.98)	1.30 (0.15, 10.4)	1.18 (0.35, 4.01)	**F**	1.77 (0.17, 19.2)	0.54 (0.06, 5.13)
	**0.09 (0.01, 0.44)**	**0.13 (0.04, 0.40)**	0.12 (0.01, 1.18)	0.75 (0.19, 2.44)	0.68 (0.06, 6.24)	0.57 (0.05, 5.80)	**G**	0.31 (0.05, 1.82)
	0.29 (0.06, 1.22)	0.45 (0.08, 2.16)	0.39 (0.05, 3.20)	2.38 (0.49, 11.9)	2.21 (0.25, 19.9)	1.87 (0.20, 16.9)	3.25 (0.55, 20.6)	**H**
**PR**	**A**	1.44 (0.78, 2.70)	1.03 (0.49, 2.23)	3.52 (2.37, 5.83)	6.47 (2.85, 15.5)	5.49 (2.39, 13.9)	6.14 (3.43, 12.3)	1.53 (0.93, 2.56)
	0.70 (0.37, 1.29)	**B**	0.72 (0.27, 1.83)	2.47 (1.44, 4.31)	4.46 (1.56, 13.7)	3.86 (1.34, 12.6)	4.29 (2.59, 7.08)	1.08 (0.62, 1.76)
	0.97 (0.45, 2.06)	1.40 (0.55, 3.71)	**C**	3.49 (1.44, 8.70)	6.34 (2.00, 21.3)	5.36 (1.69, 18.5)	6.01 (2.28, 17.0)	1.47 (0.61, 3.89)
	**0.28 (0.17, 0.42)**	**0.40 (0.23, 0.70)**	**0.29 (0.11, 0.70)**	**D**	1.89 (0.69, 4.82)	1.59 (0.57, 4.20)	1.73 (1.04, 2.98)	0.44 (0.24, 0.77)
	**0.15 (0.06, 0.35)**	**0.22 (0.07, 0.64)**	**0.16 (0.05, 0.50)**	0.53 (0.21, 1.46)	**E**	0.88 (0.50, 1.48)	0.96 (0.32, 2.88)	0.24 (0.08, 0.64)
	**0.18 (0.07, 0.42)**	**0.26 (0.08, 0.74)**	**0.19 (0.05, 0.59)**	0.63 (0.24, 1.75)	1.14 (0.68, 1.99)	**F**	1.14 (0.36, 3.40)	0.28 (0.09, 0.76)
	**0.16 (0.08, 0.29)**	**0.23 (0.14, 0.39)**	**0.17 (0.06, 0.44)**	**0.58 (0.34, 0.96)**	1.04 (0.35, 3.13)	0.87 (0.29, 2.79)	**G**	0.25 (0.13, 0.47)
	0.65 (0.39, 1.08)	0.93 (0.57, 1.62)	0.68 (0.26, 1.65)	**2.30 (1.31, 4.16)**	**4.25 (1.56, 12.9)**	**3.60 (1.32, 11.6)**	**4.03 (2.15, 7.96)**	**H**
**AAE**	**A**	2.32 (0.58, 7.84)	2.37 (0.38, 14.33)	1.31 (0.44, 4.10)	0.80 (0.13, 4.97)	0.60 (0.10, 3.42)	5.36 (0.79, 39.98)	3.95 (0.79, 18.57)
	0.43 (0.13, 1.72)	**B**	1.00 (0.12, 10.92)	0.56 (0.16, 2.63)	0.34 (0.04, 3.42)	0.25 (0.03, 2.63)	2.34 (0.44, 14.64)	1.72 (0.37, 8.81)
	0.42 (0.07, 2.64)	1.00 (0.09, 8.12)	**C**	0.56 (0.06, 4.87)	0.34 (0.02, 4.22)	0.26 (0.02, 3.20)	2.30 (0.15, 32.86)	1.68 (0.14, 18.08)
	0.76 (0.24, 2.30)	1.77 (0.38, 6.44)	1.80 (0.21, 16.06)	**D**	0.61 (0.07, 4.87)	0.46 (0.05, 3.75)	4.05 (0.63, 26.92)	3.01 (0.48, 17.77)
	1.24 (0.20, 7.90)	2.94 (0.29, 23.53)	2.94 (0.24, 41.05)	1.63 (0.21, 14.54)	**E**	0.74 (0.13, 4.50)	6.65 (0.50, 94.12)	6.70 (0.57, 68.03)
	1.68 (0.29, 9.53)	3.94 (0.38, 31.62)	3.92 (0.31, 48.92)	2.19 (0.27, 18.46)	1.36 (0.22, 7.59)	**F**	9.02 (0.67, 23.75)	0.73 (0.07, 6.92)
	0.19 (0.03, 1.27)	0.43 (0.07, 2.28)	0.43 (0.03, 6.61)	0.25 (0.04, 1.59)	0.15 (0.01, 1.99)	0.11 (0.01, 1.48)	**G**	-
	0.25 (0.05, 1.26)	0.58 (0.11, 2.72)	0.60 (0.06, 7.09)	0.33 (0.06, 2.09)	0.20 (0.02, 2.25)	0.15 (0.01, 1.74)	1.37 (0.14, 13.36)	**H**

Intervention: A: Chemotherapy; B: Ipilimumab; C: Tremelimumab; D: Nivolumab; E: Pembrolizumab 10 mg/kg; F: Pembrolizumab 2 mg/kg; G: Ipilimumab+Nivolumab; H: Ipilimumab+Chemotherapy; Outcomes: PFS: Progression Free Survival; OS: Overall Survival; CR: Complete Rate; PR: Partial Rate; AAE: All Adverse Events.

Three interventions exhibited more compelling OS results in reference to chemotherapy: Ipilimumab (HR = 0.54, 95% CrI = 0.42-0.70), Nivolumab (HR = 0.42, 95% CrI = 0.25-0.72) and Ipilimumab + Chemotherapy (HR = 0.72, 95% CrI = 0.59-0.87). Besides chemotherapy, three interventions were less effective than Ipilimumab in OS: Tremelimumab (HR = 1.62, 95% CrI = 1.21-2.18), Pembrolizumab 10 mg/kg (HR = 1.45, 95% CrI = 1.1-1.91) and Ipilimumab + Chemotherapy (HR = 1.32, 95% CrI = 1.13-1.55). Results from NMA also provided evidence that melanoma patients treated with Nivolumab were associated with a decrease in the HR in relation to those treated with Tremelimumab (HR = 0.47, 95% CrI = 0.27-0.83; Table 2, Figure 2).

**Figure 3:**
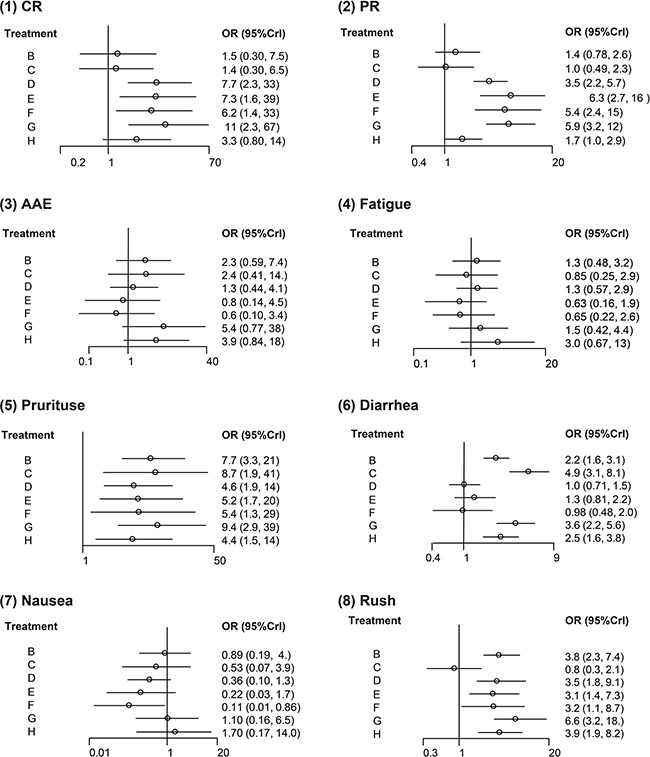
Forest plots for mixed treatment comparison of different outcomes (1) CR: complete; rate; (2) PR: partial rate; (3) AAE: all adverse events; (4) Fatigue; (5) Pruritus; (6) Diarrhea; (7) Nausea; (8) Rush.

Compared to chemotherapy, four interventions appeared to have stronger efficacy: Nivolumab (OR = 8.09, 95% CrI = 2.28-30.0), Pembrolizumab 10 mg/kg (OR = 7.36, 95% CrI = 1.54-37.9), Pembrolizumab 2 mg/kg (OR = 6.5, 95% CrI = 1.31-32.9), Ipilimumab + Nivolumab (OR = 10.8, 95% CrI = 2.29-68.3). Two interventions were significantly better than Ipilimumab with respect to CR: Nivolumab (OR = 5.58, 95% CrI = 1.61-20.0) and Ipilimumab + Nivolumab (OR = 7.43, 95% CrI = 2.51-27.3). Likewise, a few significant results were obtained from NMA with respect to the endpoint of PR. Chemotherapy were less effective than the following four interventions: Nivolumab (OR = 0.28, 95% CrI = 0.17-0.42), Pembrolizumab 10 mg/kg (OR = 0.15, 95% CrI = 0.06-0.35), Pembrolizumab 2 mg/kg (OR = 0.18, 95% CrI = 0.07-0.42) and Ipilimumab + Nivolumab (OR = 0.16, 95% CrI = 0.08-0.29). Besides, the above four interventions also exhibited stronger effectiveness than Ipilimumab as well as Tremelimumab (all OR < 1). Patients treated with Nivolumab, Pembrolizumab 10 mg/kg, Pembrolizumab 2 mg/kg and Ipilimumab + Nivolumab appeared to have more optimistic PR results compared to those treated with Ipilimumab + Chemotherapy (all OR > 1; Table 2, Figure 3).

### Comparison of adverse events among interventions

We also compared the tolerability of interventions assessed by different adverse events. Firstly, the risk of fatigue did not appear to be significantly different among melanoma patients treated with these interventions (all 95% CrI includes 1). However, patients treated with the other seven interventions were associated with an increased risk of Pruritus compared to those treated with chemotherapy: Ipilimumab (OR = 7.62, 95% CrI = 3.33-20.42), Tremelimumab (OR = 8.84, 95% CrI = 1.96-42.1), Nivolumab (OR = 4.62, 95% CrI = 1.98-13.7), Pembrolizumab 10 mg/kg (OR = 5.21, 95% CrI = 1.78-19.51), Pembrolizumab 2 mg/kg (OR = 5.40, 95% CrI = 1.35-25.58), Ipilimumab + Nivolumab (OR = 9.31, 95% CrI = 3.00-36.8) and Ipilimumab + Chemotherapy (OR = 4.45, 95% CrI = 1.53-13.4) (Table 3, Figure 3). Results from NMA also suggested that the selection of intervention might significantly affect the risk of diarrhea. Ipilimumab, Tremelimumab, Ipilimumab + Nivolumab and Ipilimumab + Chemotherapy might result in a higher risk of diarrhea compared to chemotherapy (all OR > 1). Tremelimumab might result in a higher risk of diarrhea compared to Nivolumab, Pembrolizumab 10 mg/kg, Pembrolizumab 2 mg/kg and Ipilimumab + Chemotherapy (OR > 1). Melanoma patients treated with Ipilimumab + Nivolumab or Ipilimumab + Chemotherapy were associated with an elevated risk of diarrhea compared to those treated with Nivolumab, Pembrolizumab 10 mg/kg and Pembrolizumab 2 mg/kg (all OR > 1; Table 3, Figure 3). Another safety measurement of interventions was the risk of rush. Six interventions appeared to result in an increased risk of rush: Ipilimumab (OR = 3.79, 95% CrI = 2.26-7.22), Nivolumab (OR = 3.40, 95% CrI = 1.83-8.80), Pembrolizumab 10 mg/kg (OR = 3.03, 95% CrI = 1.43-7.22), Pembrolizumab 2 mg/kg (OR = 3.06, 95% CrI = 1.10-8.36), Ipilimumab + Nivolumab (OR = 6.45, 95% CrI = 2.20-18.20) and Ipilimumab + Chemotherapy (OR = 3.78, 95% CrI = 1.85-8.05). Four interventions including Nivolumab, Pembrolizumab 10 mg/kg, Ipilimumab + Nivolumab and Ipilimumab + Chemotherapy were associated with an increased risk of rush compared to Tremelimumab (all OR > 1).

**Table 3:** Main adverse event NMA outcomes for melanoma

**Fatigue**	**A**	1.29 (0.48, 3.34)	0.85 (0.24, 3.22)	1.32 (0.56, 2.90)	0.62 (0.15, 1.88)	0.64 (0.20, 2.74)	1.47 (0.41, 4.66)	3.07 (0.68, 13.80)
	0.78 (0.30, 2.09)	**B**	0.66 (0.13, 3.29)	1.03 (0.38, 2.68)	0.49 (0.08, 1.98)	0.50 (0.12, 2.87)	1.13 (0.43, 2.81)	2.37 (0.77, 7.35)
	1.17 (0.31, 4.18)	1.51 (0.30, 7.49)	**C**	1.55 (0.32, 6.79)	0.73 (0.10, 3.64)	0.75 (0.14, 5.42)	1.71 (0.27, 9.63)	3.58 (0.49, 23.7)
	0.76 (0.34, 1.77)	0.97 (0.37, 2.63)	0.65 (0.15, 3.17)	**D**	0.47 (0.09, 1.87)	0.48 (0.12, 2.62)	1.11 (0.36, 3.37)	2.32 (0.52, 10.7)
	1.60 (0.53, 6.71)	2.05 (0.51, 11.89)	1.37 (0.27, 9.95)	2.11 (0.53, 10.9)	**E**	1.05 (0.43, 4.14)	2.36 (0.45, 14.9)	4.95 (0.77, 39.6)
	1.56 (0.36, 4.90)	2.01 (0.35, 8.49)	1.34 (0.18, 7.16)	2.07 (0.38, 8.12)	0.95 (0.24, 2.35)	**F**	2.31 (0.32, 10.9)	4.75 (0.58, 29.7)
	0.68 (0.21, 2.45)	0.88 (0.36, 2.34)	0.58 (0.10, 3.65)	0.90 (0.30, 2.82)	0.42 (0.07, 2.20)	0.43 (0.09, 3.11)	**G**	2.12 (0.50, 9.48)
	0.33 (0.07, 1.47)	0.42 (0.14, 1.31)	0.28 (0.04, 2.06)	0.43 (0.09, 1.91)	0.20 (0.03, 1.29)	0.21 (0.03, 1.71)	0.47 (0.11, 2.01)	**H**
**Pruritus**	**A**	7.62 (3.33, 20.42)	8.84 (1.96, 42.1)	4.62 (1.98, 13.7)	5.21 (1.78, 19.51)	5.40 (1.35, 25.58)	9.31 (3.00, 36.8)	4.45 (1.53, 13.4)
	**0.13 (0.05, 0.30)**	**B**	1.16 (0.18, 6.14)	0.60 (0.22, 1.75)	0.68 (0.23, 2.28)	0.71 (0.15, 3.59)	1.23 (0.45, 3.56)	0.58 (0.20, 1.60)
	**0.11 (0.02, 0.51)**	0.86 (0.16, 5.53)	**C**	0.52 (0.09, 3.65)	0.59 (0.10, 4.69)	0.61 (0.08, 5.74)	1.05 (0.17, 8.48)	0.50 (0.08, 3.32)
	**0.22 (0.07, 0.51)**	1.67 (0.57, 4.53)	1.93 (0.27, 10.6)	**D**	1.14 (0.28, 4.73)	1.18 (0.19, 6.64)	2.03 (0.61, 6.83)	0.96 (0.23, 3.42)
	**0.19 (0.05, 0.56)**	1.47 (0.44, 4.34)	1.71 (0.21, 10.4)	0.88 (0.21, 3.57)	**E**	1.03 (0.24, 4.28)	1.81 (0.39, 8.06)	0.85 (0.17, 3.41)
	**0.19 (0.04, 0.74)**	1.42 (0.28, 6.61)	1.65 (0.17, 12. 9)	0.85 (0.15, 5.15)	0.97 (0.23, 4.17)	**F**	1.75 (0.27, 11.2)	0.83 (0.13, 4.47)
	**0.11 (0.03, 0.33)**	0.81 (0.28, 2.20)	0.96 (0.12, 5.78)	0.49 (0.15, 1.64)	0.55 (0.12, 2.57)	0.57 (0.09, 3.68)	**G**	0.47 (0.11, 1.82)
	**0.22 (0.07, 0.65)**	1.71 (0.63, 5.09)	2.02 (0.30, 13.3)	1.04 (0.29, 4.31)	1.17 (0.29, 5.76)	1.21 (0.22, 7.84)	2.11 (0.55, 9.33)	**H**
**Diarrhea**	**A**	2.32 (1.67, 3.06)	5.16 (3.03, 8.37)	1.07 (0.73, 1.49)	1.34 (0.81, 2.15)	1.01 (0.45, 2.05)	3.75 (2.34, 5.68)	2.55 (1.63, 3.75)
	**0.43 (0.33, 0.60)**	**B**	2.21 (1.25, 3.91)	0.46 (0.32, 0.66)	0.58 (0.36, 0.93)	0.43 (0.20, 0.95)	1.62 (1.11, 2.34)	1.10 (0.71, 1.71)
	**0.19 (0.12, 0.33)**	**0.45 (0.26, 0.80)**	**C**	0.21 (0.11, 0.38)	0.26 (0.13, 0.54)	0.20 (0.08, 0.46)	0.73 (0.38, 1.41)	0.50 (0.26, 0.94)
	0.93 (0.67, 1.37)	**2.17 (1.51, 3.09)**	**4.78 (2.60, 8.83)**	**D**	1.27 (0.73, 2.21)	0.93 (0.41, 2.13)	3.50 (2.30, 5.32)	2.40 (1.42, 3.96)
	0.74 (0.46, 1.23)	**1.73 (1.07, 2.76)**	**3.81 (1.86, 7.59)**	0.78 (0.45, 1.38)	**E**	0.72 (0.34, 1.47)	2.79 (1.53, 4.88)	1.92 (1.01, 3.39)
	0.99 (0.49, 2.24)	**2.31 (1.06, 5.06)**	**5.09 (2.16, 12.6)**	1.08 (0.47, 2.43)	1.39 (0.68, 2.90)	**F**	3.76 (1.65, 8.57)	2.57 (1.12, 5.72)
	**0.27 (0.18, 0.43)**	**0.62 (0.43, 0.90)**	1.37 (0.71, 2.62)	**0.29 (0.19, 0.43)**	**0.36 (0.20, 0.65)**	**0.27 (0.12, 0.61)**	**G**	0.68 (0.40, 1.17)
	**0.39 (0.27, 0.61)**	0.91 (0.59, 1.40)	**2.01 (1.06, 3.85)**	**0.42 (0.25, 0.70)**	**0.52 (0.29, 0.99)**	**0.39 (0.17, 0.89)**	1.47 (0.85, 2.52)	**H**
**Rush**	**A**	3.79 (2.26, 7.22)	0.84 (0.35, 2.06)	3.40 (1.83, 8.80)	3.03 (1.43, 7.22)	3.06 (1.10, 8.36)	6.45 (3.20, 18.2)	3.78 (1.85, 8.05)
	**0.26 (0.14, 0.44)**	**B**	0.22 (0.07, 0.61)	0.90 (0.49, 2.03)	0.79 (0.37, 1.72)	0.80 (0.26, 2.15)	1.70 (0.94, 3.74)	0.99 (0.49, 1.95)
	1.19 (0.49, 2.88)	**4.45 (1.63, 13.9)**	**C**	4.03 (1.47, 16.6)	3.58 (1.15, 13.1)	3.62 (0.93, 13.7)	7.57 (2.67, 31.8)	4.51 (1.44, 14.4)
	**0.29 (0.11, 0.55)**	1.11 (0.49, 2.03)	**0.25 (0.06, 0.68)**	**D**	0.87 (0.30, 2.18)	0.89 (0.22, 2.67)	1.91 (0.88, 4.09)	1.11 (0.39, 2.53)
	**0.33 (0.14, 0.70)**	1.27 (0.58, 2.70)	**0.28 (0.08, 0.87)**	1.15 (0.46, 3.34)	**E**	1.04 (0.37, 2.31)	2.19 (0.86, 6.44)	1.28 (0.46, 3.31)
	**0.33 (0.12, 0.91)**	1.24 (0.47, 3.82)	0.28 (0.07, 1.07)	1.13 (0.37, 4.52)	0.97 (0.43, 2.67)	**F**	2.14 (0.71, 8.59)	1.25 (0.39, 4.19)
	**0.16 (0.05, 0.31)**	0.59 (0.27, 1.06)	**0.13 (0.03, 0.37)**	0.52 (0.24, 1.13)	0.46 (0.16, 1.16)	0.47 (0.12, 1.40)	**G**	0.59 (0.20, 1.35)
	**0.26 (0.12, 0.54)**	1.01 (0.51, 2.05)	**0.22 (0.07, 0.69)**	0.90 (0.40, 2.57)	0.78 (0.30, 2.18)	0.80 (0.24, 2.55)	1.70 (0.74, 5.05)	**H**
**Nausea**	**A**	0.89 (0.19, 4.10)	0.53 (0.07, 3.55)	0.36 (0.10, 1.26)	0.22 (0.03, 1.76)	0.11 (0.01, 0.86)	1.06 (0.16, 6.82)	1.67 (0.16, 13.2)
	1.12 (0.24, 5.32)	**B**	0.60 (0.05, 7.22)	0.41 (0.09, 1.82)	0.25 (0.02, 3.31)	0.12 (0.01, 1.68)	1.21 (0.29, 5.02)	1.89 (0.35, 8.61)
	1.88 (0.28, 14.0)	1.67 (0.14, 19.5)	**C**	0.68 (0.07, 7.21)	0.41 (0.03, 7.74)	0.20 (0.01, 3.75)	2.00 (0.14, 31.1)	3.18 (0.15, 57.9)
	2.75 (0.79, 10.2)	2.44 (0.55, 10.8)	1.46 (0.14, 14.31)	**D**	0.60 (0.05, 6.80)	0.30 (0.02, 3.56)	2.92 (0.55, 16.6)	4.59 (0.49, 34.8)
	4.61 (0.57, 36.5)	4.05 (0.30, 53.2)	2.45 (0.13, 40.0)	1.66 (0.15, 20.1)	**E**	0.50 (0.06, 4.37)	4.91 (0.30, 88.74	7.72 (0.34, 142)
	**9.37 (1.16, 81.1)**	8.33 (0.59, 112)	4.97 (0.27, 87.7)	3.36 (0.28, 40.7)	2.02 (0.23, 17.2)	**F**	10.1 (0.57, 170)	15.47 (0.68, 317)
	0.94 (0.15, 6.18)	0.83 (0.20, 3.44)	0.50 (0.03, 7.14)	0.34 (0.06, 1.83)	0.20 (0.01, 3.29)	0.10 (0.01, 1.75)	**G**	1.56 (0.17, 11.9)
	0.60 (0.08, 6.24)	0.53 (0.12, 2.87)	0.31 (0.02, 6.65)	0.22 (0.03, 2.05)	0.13 (0.01, 2.90)	0.06 (0.00, 1.47)	0.64 (0.08, 5.91)	**H**

Intervention: A: Chemotherapy; B: Ipilimumab; C: Tremelimumab; D: Nivolumab; E: Pembrolizumab 10 mg/kg; F: Pembrolizumab 2 mg/kg; G: Ipilimumab+Nivolumab; H: Ipilimumab+Chemotherapy.

### Assessing consistency between direct and indirect evidence

**Figure 4:**
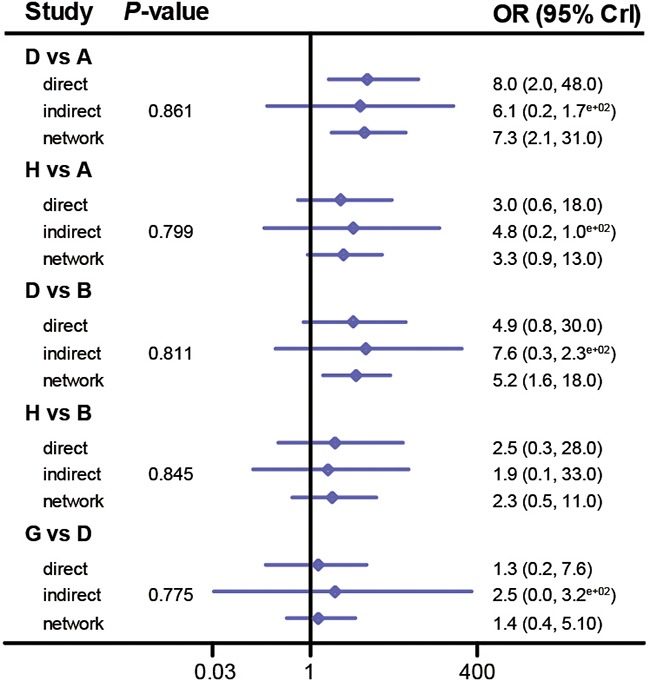
Node splitting plot of complete rate

**Figure 5:**
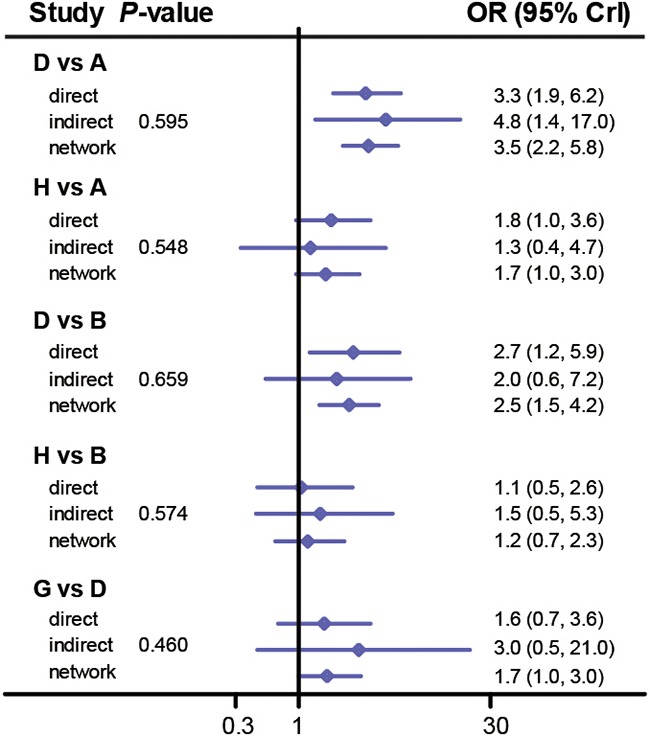
Node splitting plot of partial rate

**Figure 6:**
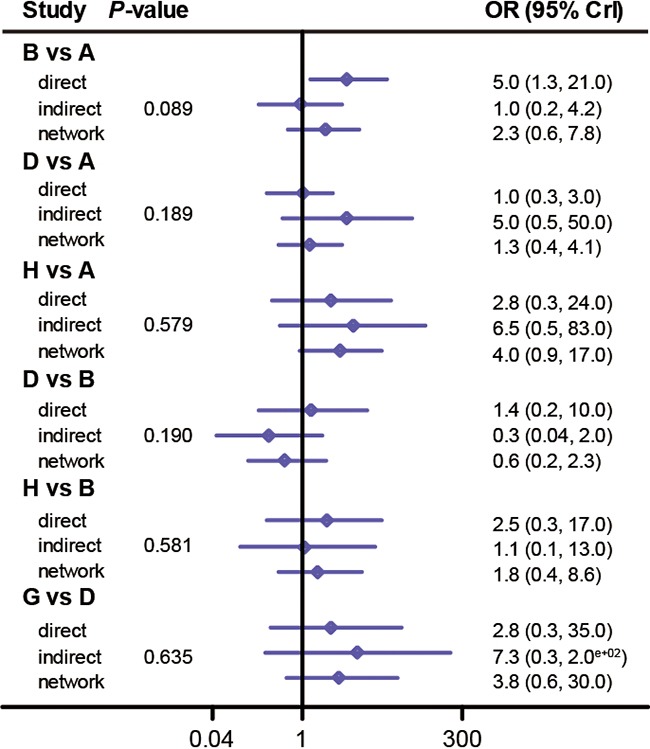
Node splitting plot of all adverse events

Since the consistency model was used in the implementation of the NMA, it was essential to assess the consistency between direct and indirect evidence for each comparison [37]. The output plot conveyed the information about the appropriateness of the consistency model which is confirmed by their corresponding *P*-values. For instance, there was no significant discrepancy between direct evidence and indirect evidence for each comparison with respect to CR (*P*-value > 0.05, Figure 4). Also, the consistency model is appropriate for comparing interventions with respect to other endpoints including PR (Figure 5), AAE (Figure 6), fatigue (Supplementary Figure S1), pruritus (Supplementary Figure S2), diarrhea (Supplementary Figure S3), nausea (Supplementary Figure S4) and rush (Supplementary Figure S5) (all *P*-value > 0.05). Therefore, we concluded that the consistency model was valid for comparing interventions with respect to the above endpoints.

### Ranking of interventions

**Figure 7:**
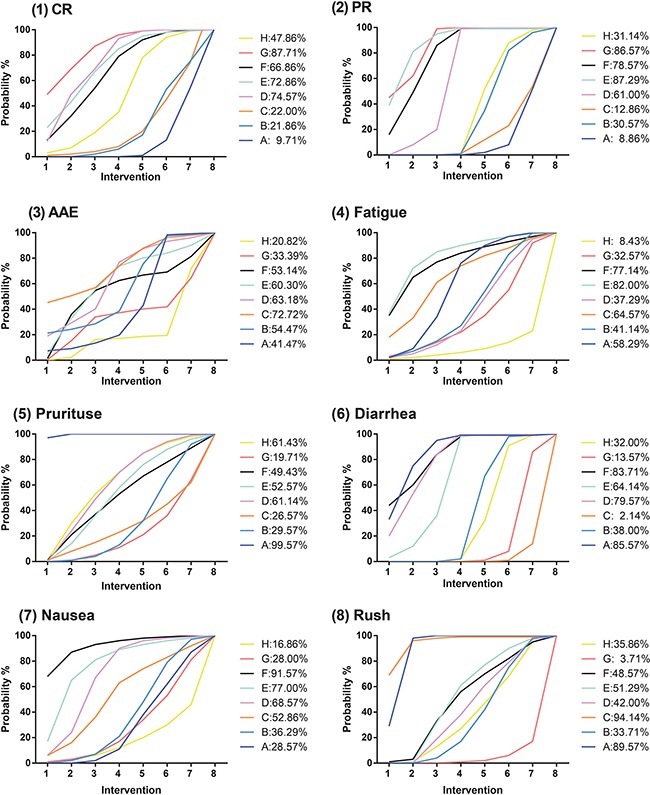
SUCRA of index except OS and PFS (1) CR: complete rate; (2) PR: partial rate; (3) AAE: all adverse events; (4) Fatigue; (5) Pruritus; (6) Diarrhea; (7) Nausea; (8) Rush.

One advantage of carrying out a NMA with the Bayesian framework is its ability to produce ranking probabilities which can be used to discriminate interventions with respect to each endpoint. As suggested by the accumulative ranking probability plots and SUCRA values (Table 4, Figure 7), Ipilimumab + Nivolumab exhibited the highest SUCRA with respect to PFS (0.929) and CR (0.857). The standard chemotherapy appeared to have relative weak effectiveness as indicated by lower SUCRA values for PFS, OS, CR and PR whereas such a disadvantage was compensated by its higher SUCRA values with respect to AAE, fatigue, pruritus, diarrhea and rush. On the other hand, Pembrolizumab 10 mg/kg or 2mg/kg appeared to balance between effectiveness and safety since its enhanced efficacy due to increase in dose was not compensated by its increased toxicity. The combined therapy of Ipilimumab + Nivolumab appeared to have higher SUCRA values with respect to PFS, CR and PR. However, such an enhancement was offset by its low SUCRA values with respect to several adverse events.

**Table 4:** SUCRA results for eight intervention outcomes in treatments of melanoma

Intervention	PFS	OS	CR	PR	AAE	Fatigue	Pruritus	Diarrhea	Rush	Nausea
Chemotherapy	0.042	0.053	0.097	0.089	0.643	0.583	0.996	0.856	0.896	0.286
Ipilimumab	0.414	0.839	0.219	0.306	0.143	0.411	0.296	0.380	0.337	0.363
Tremelimumab	0.718	0.258	0.220	0.129	0.393	0.646	0.266	0.021	0.941	0.529
Nivolumab	0.820	0.941	0.746	0.610	0.500	0.373	0.601	0.796	0.420	0.686
Pembrolizumab 10 mg/kg	0.321	0.380	0.729	0.873	0.964	0.820	0.526	0.641	0.513	0.770
Pembrolizumab 2mg/kg	0.456	0.494	0.669	0.786	0.893	0.771	0.494	0.837	0.486	0.916
Ipilimumab+Nivolumab	0.929		0.857	0.866	0.000	0.326	0.197	0.136	0.037	0.280
Ipilimumab+Chemotherapy	0.301	0.534	0.479	0.341	0.464	0.084	0.614	0.320	0.359	0.169

Outcomes: PFS: Progression Free Survival; OS: Overall Survival; CR: Complete Rate; PR: Partial Rate; AAE: All Adverse Events.

## DISCUSSION

The effectiveness of chemotherapy for managing melanoma patients is quite debatable due to the heterogeneity of tumors. Most melanoma patients are treated with either dacarbazine (DTIC) or temozolomide which contributes to a low objective response rate of less than 15% [38]. The development of targeted therapy aimed at gene mutations and aberrant cell signaling pathways appears to somewhat complement the standard chemotherapy. Melanoma patients treated with targeted therapy benefit from substantial increase in the overall response rate as well as extended survival [39–41]. Unfortunately, most melanoma patients develop resistance to targeted therapies and eventually experience metastatic tumor lesions [39, 40]. On the other hand, modest and long-lasting responses have been shown by antibodies which target CTLA-4 or PD-1. Unlike targeted therapy, more modest but durable responses were observed with patients treated with immune checkpoint inhibitors. However, such a durable response is not reflected by all patients due to significant heterogeneity in lymphocyte infiltration [38].

Immune checkpoint blockade antibodies targeting CTLA-4 and PD-1 have become a major breakthrough in cancer treatments for some patients with advanced melanoma. Compared to chemotherapy and molecularly targeted therapy, immune checkpoint therapy is able to provide more durable clinical response through the induction and activation of tumor-specific cytotoxic T cells [42]. It is well known that immune checkpoints have two important roles: maintenance of self-tolerance as well as regulating the amplitude and duration of T cells. Theoretically, immune checkpoint blockade antibodies that block CTLA-4 and PD-1 are able to restore and enhance cytotoxic T cell responses against tumors which exhibit resistance to chemotherapy [42].

Ipilimumab is a human monoclonal antibody that blocks CTLA-4 and was considered as the first option for advanced metastatic melanoma based on phase II and III trials [22, 43]. Our study not only verified that Ipilimumab exhibits enhanced efficacy compared with chemotherapy but also concluded that the combined intervention of Ipilimumab + Nivolumab is more effective than Ipilimumab monotherapy. Our results are supported by two randomized trials which suggest that the combined intervention of Ipilimumab + Nivolumab is recommended for patients with unresectable metastatic melanoma since they appear to have complementary activity in this disease [31]. Another phase II study in which patients with BRAF wild-type melanoma are included indicated that the combined strategy of Ipilimumab + Nivolumab is far more effective than Ipilimumab monotherapy since the corresponding CR rate for two treatment group were 22% and 0%, respectively [33]. Therefore, introducing Nivolumab into Ipilimumab may enhance the effectiveness of Ipilimumab monotherapy.

Tremelimumab is another fully humanized monoclonal antibody targeting CTLA-4. Despite that some promising results have been observed in Phase I or II trials, Tremelimumab in phase III studies does not exhibit significant results for melanoma patients [10]. This may be explained by the fact that Tremelimumab binds to FcγR with lower affinity, which results in less effective blocking of CTLA-4 to T-cells [10]. As suggested by the corresponding ranking probabilities, Tremelimumab appears to be more effective than chemotherapy in PFS, OS, CR and PR. However, such a strength may be offset by its increased toxicity since patients treated with Tremelimumab are more likely to experience adverse events such as pruritus and diarrhea compared to those treated with chemotherapy. Monoclonal antibodies targeting CTLA-4 result in toxicities related to organ-specific inflammatory processes [44]. Therefore, higher doses may result in both higher likelihood antitumor responses and toxicity. Nevertheless, our study does not enable us to perform a stratified analysis by dose and such a mechanism should be thoroughly studied. However, it is still a challenging task for researchers to simultaneously assess how different dosages of melanoma interventions contribute to different levels of toxicity, because it may require biopsies of organs before and after applying interventions to patients, which is only feasible in the intestinal tract or the skin [44].

PD-1 is another checkpoint molecule that has a significant role in T-cell regulation and it suppresses the activity of T-cells in peripheral tissues when an inflammatory response is triggered [45]. Furthermore, PD-1 is a 55 kDa type I transmembrane protein encoded by the *PDCD1* gene. This protein is comprised of several elements, including a extracellular immunoglobulin (Ig) domain, a transmembrane domain and an intracellular domain that contains phosphorylation sites [45]. Nivolumab is a fully human monoclonal IgG4 antibody that binds PD-1 with high affinity and it also prevents interaction with PD-L1 and PD-L2 which are two known ligands of PD-1. As suggested by in vitro assays, Nivolumab is able to increase both antigen-specific T-cell responses and cytokine production [46]. Moreover, Nivolumab is administered intravenously over a period of 60 minutes and its serum half-life approximately ranges from 12 days to 20days depending on the corresponding dosage [45]. Another feature of Nivolumab is its linear pharmacokinetics which is able to provide durable responses in patients with a wide range of advanced malignancies including melanoma, which has been verified in phase I trials [47]. Nivolumab is generally well-tolerated by the majority of patients. The most common adverse events associated with Nivolumab include low-grade fatigue, musculoskeletal side effects, decreased appetite, nausea, diarrhea, rash and pruritus [48]. In our study, patients treated with Nivolumab are associated with a significant increase in efficacy compared with those treated with chemotherapy. Nevertheless, this trend is accompanied with an increase in the likelihood of adverse events such as fatigue, pruritus, diarrhea and rush.

Our study not only compared different interventions for melanoma patients but also investigated how different dosage of Pembrolizumab affects its efficacy and tolerability. Currently, the recommended dose of Pembrolizumab for advanced melanoma patients is 2mg/kg and it is administered via intravenous infusion over 30min every three weeks [15]. As suggested by a keynote trial in which a total of 655 patients were allocated to different treatment groups, Pembrolizumab is able to provide durable antitumor activity for patients who are refractory to Ipilimumab [29, 49]. Another randomized clinical trial which compared Pembrolizumab with chemotherapy indicated that melanoma patients treated with Pembrolizumab exhibit more desirable PFS status [50]. More importantly, a large phase III suggested that both PFS and OS status for advanced melanoma patients treated with Pembrolizumab were significantly prolonged compared to those treated with Ipilimumab [35, 50]. The above conclusion is consistent with the results obtained from our study which concluded that melanoma patients treated with Pembrolizumab have significantly higher CR and PR than those treated with chemotherapy or Ipilimumab. Moreover, autoimmune complications resulted from Pembrolizumab are fewer and less severe as compared to those treated with antibodies targeting CTLA-4 [50]. This conclusion is supported by our analysis since the corresponding SUCRA values of Pembrolizumab (10 mg/kg or 2mg/kg) with respect to adverse events are significantly higher than those of the two CTLA-4 antibodies (Ipilimumab, Tremelimumab). Although there appears to be slight difference in the SUCRA values between different doses of Pembrolizumab, pooled statistics such as HR/OR between them do not differ significantly with respect to all endpoints. Generally, increasing the dosage of Pembrolizumab may not have significant effect on its effectiveness nor tolerability. A number of studies have been designed to discover the optimal dose of Pembrolizumab, but none of them showed significant results and therefore 2 mg/kg is still considered as the recommended dose for melanoma patients. In addition, it is also recommended that thyroid function should be routinely monitored in patients who are treated with Pembrolizumab.

However, only a portion of patients respond to immune checkpoint therapy. Identifying patients who are most likely to respond to each immune checkpoint therapy is critical to the selection of interventions. However, this task cannot be achieved by simply comparing the effectiveness and tolerability of different interventions since randomized trials are usually carried out in different populations. On top of that, we do not have any knowledge about whether prior therapies had been applied to these melanoma patients and such a limitation may consequently affect the effectiveness and tolerability of immune checkpoint therapies. Also, formalized stopping rules which determine the corresponding treatment duration for each immune checkpoint therapy are not provided in most of the studies and hence it is challenging to determine the optimal duration for each immune checkpoint therapy. It is likely that the selection of treatments may be influenced by treatment schedule and costs which may distort the effect of randomization in clinical trials.

Overall, our study provides solid evidence that several immune checkpoint therapies or combined interventions may be more effective than chemotherapy for managing melanoma patients. However, chemotherapy appears to be more tolerable than these combined strategies since patients are less likely to experience adverse events. In the future, we encourage researchers to conduct ongoing clinical trials in which confounding factors are controlled in order to prevent biased results.

## MATERIALS AND METHODS

### Search strategy

We began our research with the formulation of a comprehensive searching strategy. A thorough literature search was conducted in PubMed, Embase and Cochrane Library. Firstly, we searched previous systematic reviews and NMA in order to ensure that this topic has not been carried out in the current literature. The entire searching strategy was carried out by two independent researchers without limitations to identify studies about pharmacological interventions for melanoma. Search results were compared and the differences were resolved by discussion. Disagreements after discussion were viewed and validated by a third independent researcher. Additional articles were obtained by scanning reference lists of relevant studies. The entire research question was break down into small pieces so that the scope of the question can be clearly defined.

### Inclusion criteria and screen of individual studies

A few guidelines were created for screening eligible studies: 1) study design must be randomized clinical trials with subjects older than 18 years; 2) at least one comparison was made among interventions including chemotherapy, Ipilimumab, Tremelimumab, Nivolumab, Pembrolizumab (10 mg/kg or 2 mg/kg), Ipilimumab + Nivolumab and Ipilimumab + Chemotherapy; 3) At least one of the following endpoints were evaluated by the study, including progression free survival (PFS), overall survival (OS), complete response (CR), partial response (PR), all adverse events (AAE), fatigue, pruritus, diarrhea, rush and nausea; 4) the corresponding data required for NMA must be available within the study. We used PICOS approach in this study and the detailed PICOS criteria we followed are specified in Supplementary Table S2. Individual studies that does not comply with the selection criteria were excluded.

### Data extraction and quality assessment

Data extraction and synthesis was conducted by two reviewers independently by using a data extraction spreadsheet. The following data were extracted and recorded from eligible studies: author, intervention, dosage, study sample size, average age of subjects, percentage of males, metastasis status and the corresponding endpoints. Moreover, interventions are carefully selected in order to form a closed loop for the implementation of NMA. Endpoints were clearly defined in the following ways so that selection bias can be minimized: PFS (time between the start of treatment and documented disease progression or death due to any cause); OS (time between the start of treatment and death due to any cause); CR (disappearance of all target lesions in response to treatment); PR (more than 30% decrease in the sum of diameters of target lesions in response to treatment).

The quality of included randomized controlled trials was evaluated by the Jadad scale [51]. Randomization, blinding and withdrawal were used as three scoring items.

### Statistical analysis

We carried out the NMA by using the Bayesian conceptual framework which incorporates a prior probability distribution, a likelihood function based on data and a posterior probability distribution that combines the first two elements [52]. Comparisons between interventions with respect to OS and PFS were achieved by computing the statistics of hazard ratio (HR) with 95% confidential intervals (CrIs). The odds ratio (OR) with 95% CrIs was used to compare interventions with respect to other endpoints. Furthermore, the node-splitting method is adopted to evaluate the extent of consistency between direct and indirect evidence within the network [37]. The ranking of interventions with respect to each endpoint is performed by using the corresponding surface under the cumulative ranking area (SUCRA) introduced by Salanti et al [53]. The SCURA is a useful numerical summary and allows to identify the probability of being best, being worst etc. The larger the SUCRA value, the better the rank of the treatment. Between-study heterogeneity which may arise from different sources of study variability is assessed by the Cochran's *Q* test and the degree of heterogeneity is quantified by the statistics of *I^2^*. Finally, publication bias is visually assessed by using the funnel plot in which asymmetry pattern may provide evidence for publication bias. All statistical procedures were implemented using WinBugs (MRC Biostatistics Unit) and R software (Version 3.2.4) with package ‘gemtc’ (version 0.8).

## SUPPLEMENTARY MATERIALS FIGURES AND TABLES



## Abbreviations

NMA: network meta-analysis
OS: overall survival
PFS: progression free survival
CR: complete response
PR: partial response
HR: hazard ratio
OR: odds ratio
CTLA-4: cytotoxic T-lymphocyte antigen-4
PD-1: programmed death 1
SUCRA: surface under the cumulative ranking area
