# Supplementary material for: Pharmacological interventions for melanoma: Comparative analysis using bayesian meta-analysis
Source: Oncotarget. 2016 Oct 13;7(49):80855–71. doi: 10.18632/oncotarget.12644 (PMC5348360; doi:10.18632/oncotarget.12644)
Supplement: Supplementary file 1 [file oncotarget-07-80855-s001.pdf]

Pharmacological interventions for melanoma: Comparative analysis using Bayesian meta-analysis

SUPPLEMENTARY FIGURES AND TABLES

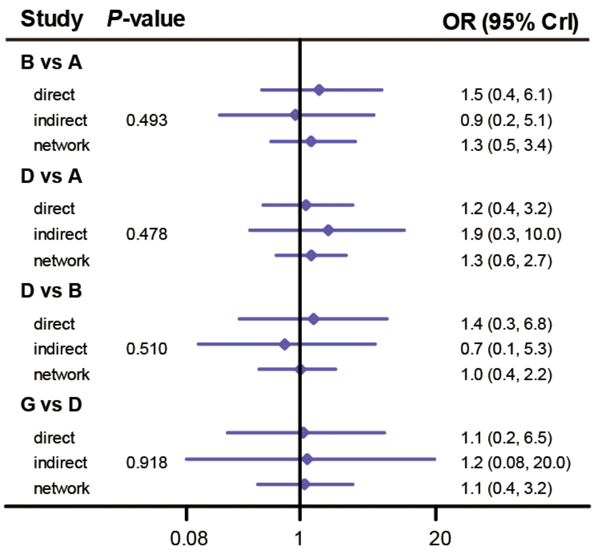

Supplementary Figure S1: Node splitting plot of fatigue.

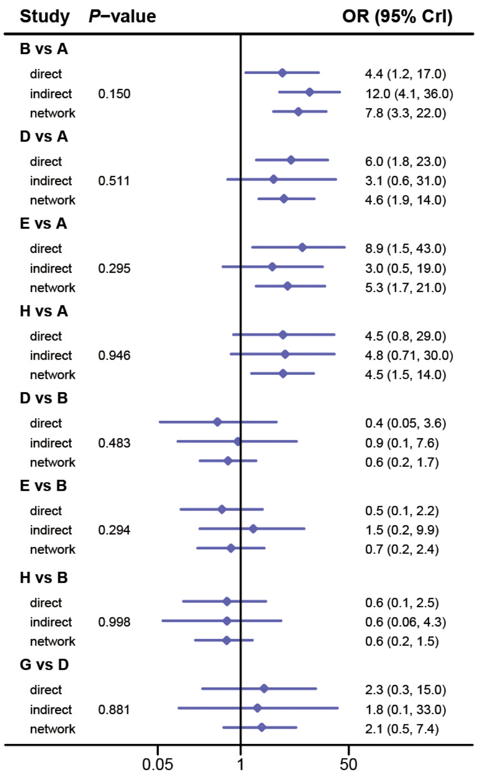

Supplementary Figure S2: Node splitting plot of pruritus.

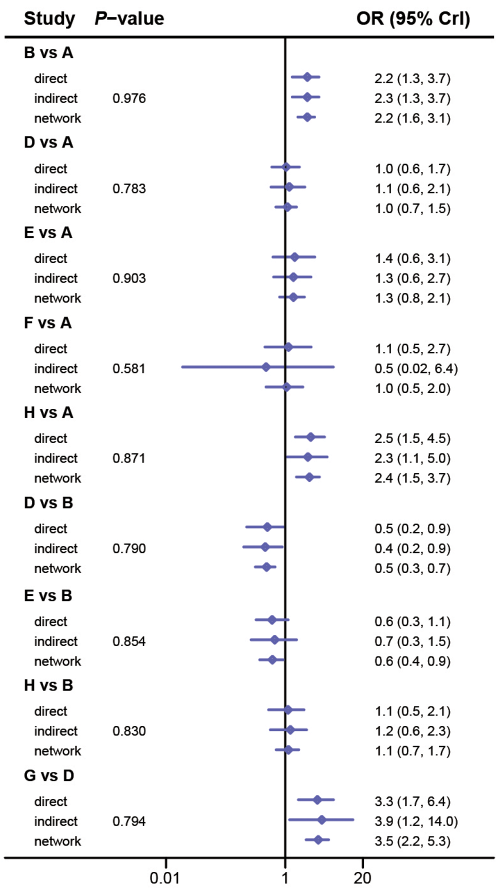

Supplementary Figure S3: Node splitting plot of diarrhea.

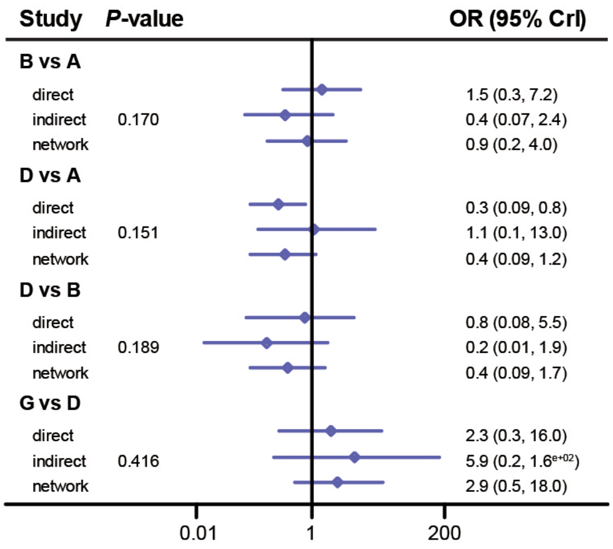

Supplementary Figure S4: Node splitting plot of nausea.

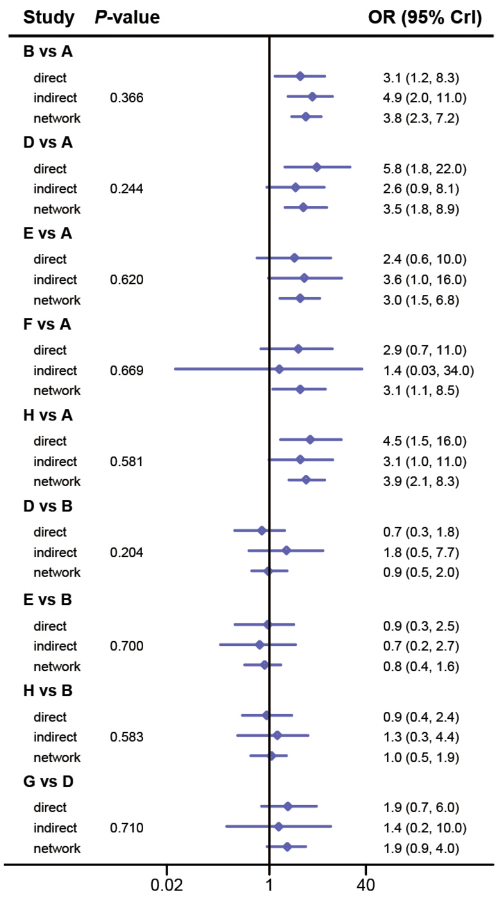

Supplementary Figure S5: Node splitting plot of rush. For all: Outcomes: PFS: Progression Free Survival; OS: Overall Survival; CR: Complete Rate; PR: Partial Rate; AAE: All Adverse Events. Intervention: A: Chemotherapy; B: Ipilimumab; C: Tremelimumab; D: Nivolumab; E: Pembrolizumab 10 mg/kg; F: Pembrolizumab 2 mg/kg; G: Ipilimumab + Nivolumab; H: Ipilimumab + Chemotherapy.

Supplementary Table S1: Jadad scale of 19 included studies

Author, Year	Randomization	Blinding	Withdrawals
Weber, 2015	2	0	1
Robert, 2015	2	0	1
Robert, 2015	2	1	0
Ribas, 2015	2	2	1
Postow, 2015	2	1	0
Miao, 2015	2	0	0
Larkin, 2015	2	1	1
Eggmont, 2015	2	2	0
Robert, 2014	2	2	1
Hodi, 2014	2	0	1
Ribas, 2013	2	0	1
Millward, 2013	1	1	1
Robert, 2011	2	2	1
Hersh, 2011	2	0	1
Hamid, 2011	2	2	1
Wolchok, 2011	2	2	1
Weber, 2009	2	2	1
Camacho, 2009	2	0	1
Ribas, 2005	1	1	1

Randomization: Was the study described as randomized?
Blinding: Was the study described as double blind?
Withdrawals: Was there a description of withdrawals and dropouts?
Each question was to be answered with either a yes or a no. Each yes would score a single point, each no zero points; there were to be no fractional points. The method of randomization was described in the paper, and that method was appropriate (1 extra point in randomization part). The method of blinding was described, and it was appropriate (1 extra point in blinding part).

Supplementary Table S2: PICOS criteria for inclusion and exclusion of studies

Parameter	Inclusion criteria	Exclusion criteria
Patients	Adults ≥ 18 years old with melanoma	Patients under 18 years old
Intervention	Chemotherapy, ipilimumab, tremelimumab, nivolumab, pembrolizumab (10 mg/kg or 2 mg/kg), ipilimumab + nivolumab or ipilimumab + chemotherapy	None of the interventions included
Comparison	Any comparison pair among the interventions	Only comparisons with other unwanted interventions
Outcomes	At least one of the following outcomes:Efficacy outcomes: progression free survival (PFS), overall survival (OS), complete response (CR), partial response (PR)Safety outcomes: all adverse events (AAE), fatigue, pruritus, diarrhea, rush, nausea	None of the outcomes includedData of outcomes not available for meta-analysis
Study design	Randomized controlled trials	Any other type of studies

PICOS: patients, interventions comparison, outcomes and study design.
